# Ultrasound-Guided Transoral Drainage of a Challenging Parapharyngeal Abscess: A Case Report

**DOI:** 10.7759/cureus.94168

**Published:** 2025-10-09

**Authors:** Maria C Michali, Lentiona Basiari, Ioannis D Komnos, Dimitra G Simou, Georgios V Psychogios

**Affiliations:** 1 Department of Otorhinolaryngology, Head and Neck Surgery, University Hospital of Ioannina, Ioannina, GRC

**Keywords:** drainage, intraoperative ultrasound, lateral neck approach, parapharyngeal abscess, transoral approach, ultrasound guided

## Abstract

Parapharyngeal space infections are uncommon but associated with significant morbidity and mortality. Early surgical drainage remains the mainstay of treatment; however, the optimal surgical approach continues to be debated. Management typically involves either an external (transcervical) approach or a transoral route.

We present the case of a 43-year-old woman with a deep parapharyngeal abscess unresponsive to empirical antibiotic therapy who presented with dysphagia, fever, and left cervical swelling. Computed tomography (CT) confirmed the presence of the abscess. Ultrasound-guided intraoral drainage was performed, followed by intravenous antibiotic therapy. The patient had an uneventful recovery and was discharged on the sixth hospital day. Parapharyngeal abscess is the second most common deep neck space infection after peritonsillar abscess. Accurate diagnosis relies on clinical evaluation and radiologic imaging, with CT considered the gold standard for both initial diagnosis and ongoing monitoring.

## Introduction

Infections of the deep neck spaces, including the parapharyngeal and retropharyngeal spaces, are relatively uncommon and can lead to serious, potentially life-threatening complications. Deep neck abscesses, such as peritonsillar, retropharyngeal, and parapharyngeal abscesses, typically arise from the spread of infection from adjacent structures, most commonly from infected tonsils, with peritonsillar abscess being the most frequent [1].

Parapharyngeal abscesses often occur as secondary complications of peritonsillar infections and, less commonly, as a result of acute tonsillitis [2,3]. The close anatomical relationship between the peritonsillar and parapharyngeal spaces facilitates the spread of infection [4]. If not promptly diagnosed and managed, these infections can result in severe complications, including airway obstruction, descending mediastinitis, internal carotid artery rupture due to mycotic aneurysm, jugular vein thrombosis, Lemierre’s syndrome, and Horner’s syndrome. Initial management focuses on airway stabilization and the administration of broad-spectrum intravenous antibiotics [2,5].

Surgical drainage is often required in addition to antibiotic therapy. The two main surgical approaches for parapharyngeal abscess drainage are the transoral and transcervical routes. Transoral drainage is generally preferred for medially located, localized abscesses, while the transcervical approach is indicated for laterally positioned or multiloculated infections. However, the choice of surgical technique remains a topic of debate, and no standardized consensus exists [6-9].

Advances in imaging have improved early diagnosis. Computed tomography (CT) remains the gold standard, but ultrasound and magnetic resonance imaging (MRI) also play essential roles in assessment and monitoring [10,11]. Ultrasound is a noninvasive, cost-effective, and radiation-free modality, making it particularly useful in pediatric and pregnant patients. Its real-time imaging capability can also assist in guiding drainage procedures.

In this report, we describe the successful use of intraoperative ultrasound-guided transoral drainage in a patient with a challenging parapharyngeal abscess, highlighting this approach as a viable alternative to conventional transcervical drainage.

## Case presentation

A 43-year-old female presented to the Emergency Department with a six-day history of dysphagia, odynophagia, and fever reaching 39°C. She had been treated at another hospital with clarithromycin and methylprednisolone without clinical improvement.

On physical examination, a firm, tender swelling was noted in the left cervical region. Additionally, a rash on the upper lip prompted dermatologic consultation, and topical mupirocin was initiated for suspected impetigo. Oropharyngeal examination revealed uvular edema. Flexible endoscopy showed mucosal edema over the left arytenoid cartilage and the left glossoepiglottic fold. Laboratory examinations showed a white blood cell (WBC) count of 11 × 10³/μL, with 90.7% neutrophils and 97 mg/L C-reactive protein (CRP; normal: <6 mg/L).

An urgent CT scan of the neck revealed a left-sided parapharyngeal abscess measuring 1.9 × 1.6 × 2.7 cm. Edema of the left lateral pharyngeal wall and epiglottis was also observed, along with reactive cervical lymphadenopathy. The abscess originated at the level of the lower pole of the left palatine tonsil, extending to the posterior aspect of the hyoid bone. It extended anteriorly toward the internal and external carotid arteries, just above the carotid bifurcation, and remained medial to the internal jugular vein.

**Figure 1:**
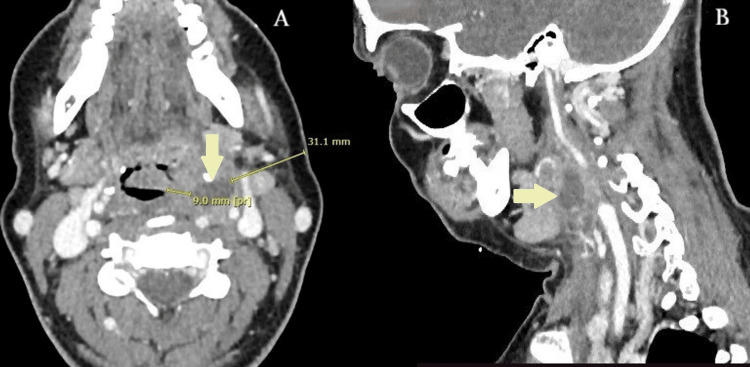
CT scan of the neck demonstrating the parapharyngeal abscess: (A) axial view; (B) sagittal view. CT, Computed tomography

A DICOM (Digital Imaging and Communications in Medicine) imaging software was employed to measure the distance between the lateral pharyngeal wall and adjacent major vessels. At the level of the epiglottis, the abscess was found approximately 9 mm deep from the lateral pharyngeal wall and extended into the parapharyngeal space towards the neck. The closest perpendicular distance from the skin was 31.1 mm. Given the abscess’s medial location and its proximity to major vessels, the surgical team debated between a transoral and transcervical approach (Figure 1).

**Figure 2:**
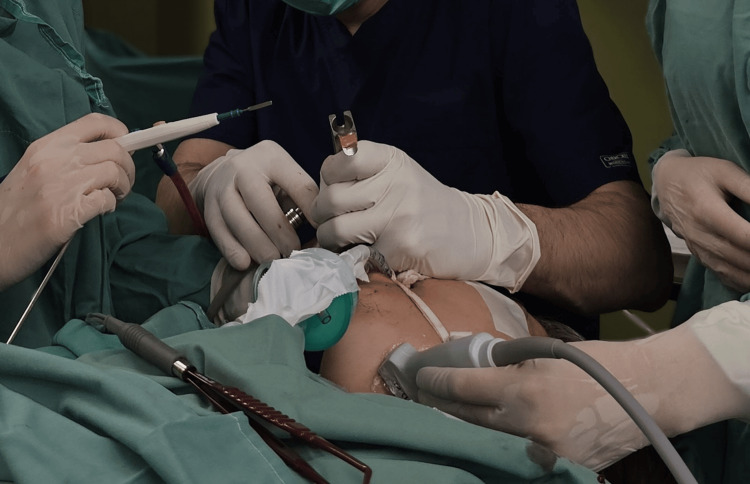
Ultrasound imaging used for localization of the abscess cavity.

**Figure 3:**
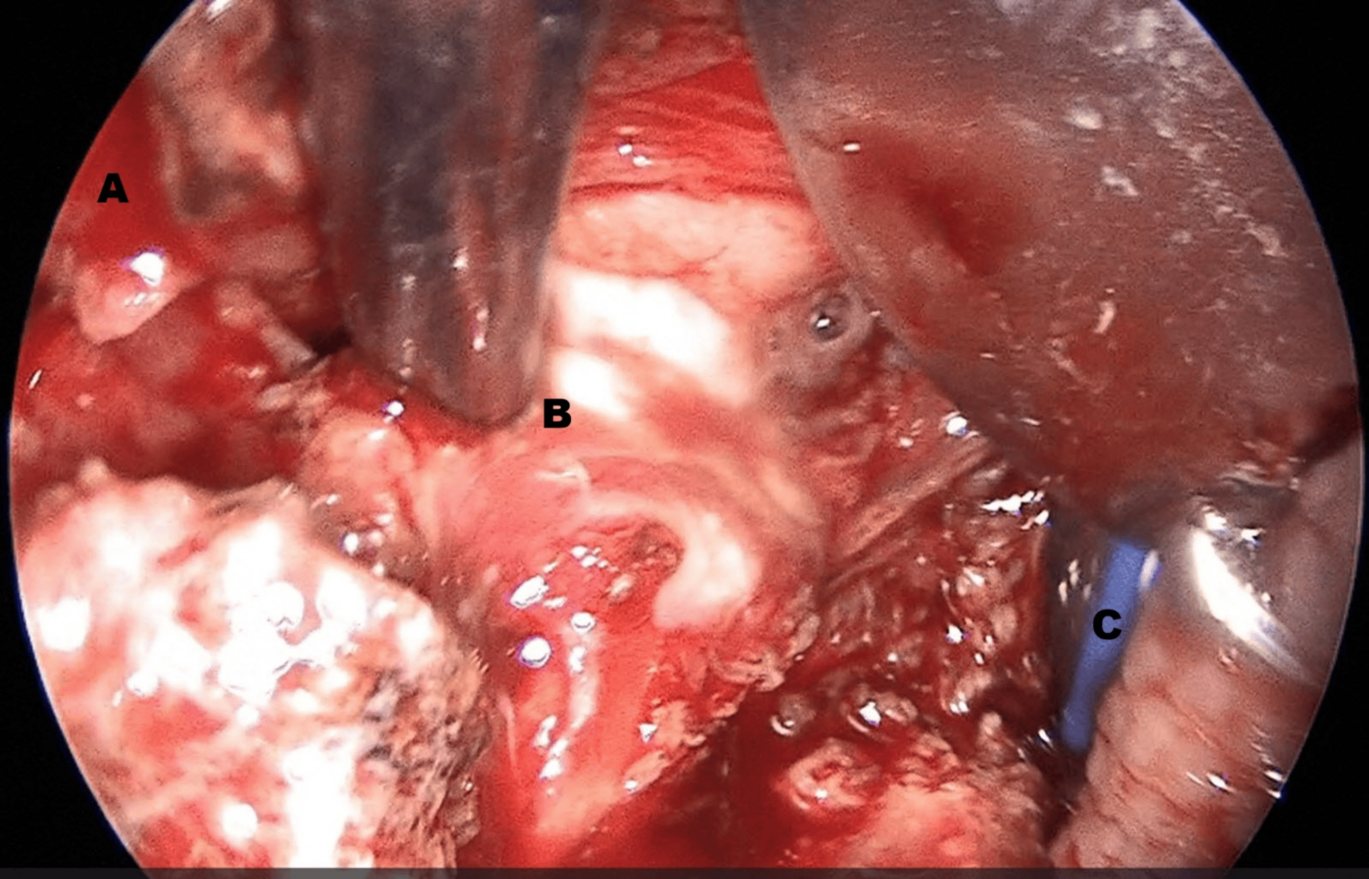
Transoral incision and drainage of abscess with transcervical ultrasound guidance. A) Area previously occupied by the lower pole of the left palatine tonsil following tonsillectomy. B) The tip of the atraumatic forceps during the evacuation of purulent material from the abscess cavity. C) The endotracheal tube, visible within the airway.

A transoral approach was ultimately selected. Initially, a left tonsillectomy was performed to enhance surgical access through the pharyngeal constrictor muscle and reduce the vertical distance to the abscess cavity. Localizing the abscess proved challenging, using an atraumatic Overholt clamp passed through the pharyngeal constrictor muscle at the site of the previously excised lower pole of the left palatine tonsil. Intraoperative ultrasound was then employed to accurately identify the abscess cavity. Using a Kleinsasser laryngoscope and suction, the cavity was accessed; atraumatic forceps were used to enlarge the opening and facilitate drainage of the purulent material. Pus was aspirated for culture, followed by multiple irrigations to ensure thorough evacuation of the abscess cavity. A feeding tube was placed to protect the surgical site and reduce hemorrhage risk, given the proximity to major vascular structures. The surgical field was thoroughly irrigated, and the patient was transferred to the recovery unit (Figures 2, 3).

Microbiological analysis of the purulent material identified *Streptococcus parasanguinis* and coagulase-negative staphylococci (CoNS). The patient received intravenous ceftriaxone and metronidazole. Her postoperative course was uneventful, with resolution of symptoms within 24 hours. A follow-up CT scan on postoperative day 4 showed complete radiological resolution of the abscess. She was discharged on postoperative day 6, with improved inflammatory markers (CRP 10 mg/L and WBC 7 × 10³/μL).

## Discussion

Parapharyngeal infections remain potentially dangerous due to their proximity to vital structures and the risk of severe complications. Advances in diagnostic imaging and surgical techniques have significantly improved the early identification and management of deep neck space infections, including parapharyngeal abscesses. Early diagnosis facilitates timely therapeutic intervention, reducing morbidity and preventing potentially life-threatening complications [12-14].

Treatment options include intravenous antibiotics alone in selected stable patients or surgical drainage in cases with well-formed abscesses [13,14]. Both transcervical and transoral approaches are utilized for parapharyngeal abscess drainage, with a recent trend favoring the transoral route in appropriately selected cases [13,14].

The transoral approach is generally considered suitable for localized abscesses, particularly those located medially to the great vessels or near the skull base [15]. However, one limitation of the transoral route is the lack of direct visualization of the abscess cavity. Dissection through the superior pharyngeal constrictor muscle carries a risk of injury to major cervical vessels and may complicate accurate abscess localization [13]. In contrast, the transcervical approach may offer better exposure for extensive or multiloculated collections but requires a more invasive incision and is associated with a higher risk of damaging the carotid sheath, especially due to the anatomical barrier of the mandible [12].

Other surgical routes to the parapharyngeal space, including the parotid and transmandibular (split-mandible) approaches, have been described in the context of large benign or malignant parapharyngeal tumors to facilitate en bloc removal and minimize the risk of capsule rupture [16]. However, these extensive techniques are not indicated in infectious cases, where the goal is safe and complete evacuation of purulent material rather than en bloc excision [16].

Imaging plays a central role in the evaluation and management of parapharyngeal abscesses. CT is considered the gold standard due to its high sensitivity, rapid acquisition, and wide availability. It provides critical information about abscess size, location, and proximity to surrounding structures, guiding the choice of surgical approach [17,18]. MRI offers superior soft tissue contrast and avoids ionizing radiation, making it preferable in children, pregnant women, and for follow-up imaging, although it is less suitable in emergencies due to longer acquisition times and reduced accessibility [17,18].

Ultrasound is less commonly used for initial diagnosis due to limited penetration depth, especially in deep neck infections. However, it can be valuable as a guidance tool for needle aspiration or during surgery. Duque et al. reported its successful use in guiding percutaneous aspiration and detecting fluid collections during the surgical exploration of parapharyngeal space abscesses in pediatric patients [18]. Additionally, Delides et al. demonstrated the intraoperative use of ultrasound to assist transoral drainage by helping localize the abscess cavity [19].

Although cervical ultrasound was used intraoperatively in our patient, transoral ultrasound employing a transvaginal probe has also been proposed as an alternative technique for evaluating deep parapharyngeal or retropharyngeal collections [20]. This approach provides high-resolution, near-field imaging through the oral cavity, which may assist in delineating abscess boundaries. However, its practical application during drainage remains limited, as the probe occupies most of the oral aperture, making it technically difficult to introduce instruments for aspiration or to dissect muscle fibers to access the abscess cavity [20].

In our case, intraoperative cervical ultrasound proved to be a valuable adjunct during transoral drainage. Given the deep and medial position of the abscess and its proximity to critical vascular structures, ultrasound enabled precise and safe localization of the abscess cavity. This facilitated accurate instrument placement, minimized the risk of vascular injury, and allowed real-time confirmation of adequate drainage. Our findings support the potential utility of intraoperative ultrasound as a guiding tool in complex parapharyngeal abscess cases managed via the transoral approach.

## Conclusions

Parapharyngeal space abscesses pose a significant clinical challenge due to the complex regional anatomy and potential for serious complications. Accurate diagnosis and timely surgical intervention are critical to achieving optimal patient outcomes. This case highlights the utility of intraoperative cervical ultrasound as an adjunct during transoral drainage of a challenging parapharyngeal abscess. Ultrasound guidance allowed for precise localization of the abscess cavity, minimized the risk of injury to adjacent vital structures, and facilitated effective evacuation. Further studies, involving larger patient cohorts, are needed to validate these observations and support the development of standardized protocols for ultrasound-guided transoral drainage in deep neck infections.
